# Supplementary material for: Drug repurposing for aging research using model organisms
Source: Aging Cell. 2017 Jun 16;16(5):1006–15. doi: 10.1111/acel.12626 (PMC5595691; doi:10.1111/acel.12626)
Supplement: Supplementary file 7 — Data S1 Zip‐Archive of all report cards. [file ACEL-16-1006-s007.zip › RC_DTF.pdf]

DTF

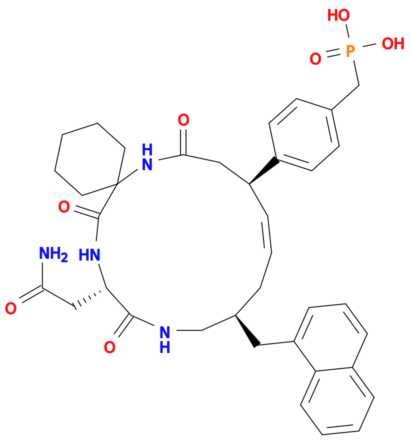

Database identifiers

ChEMBLCompound	CHEMBL291678
DrugBank	DB03276

Ranking

	Rank	Score
Drosophila	314/697	0.505
C. elegans	561/591	0.015

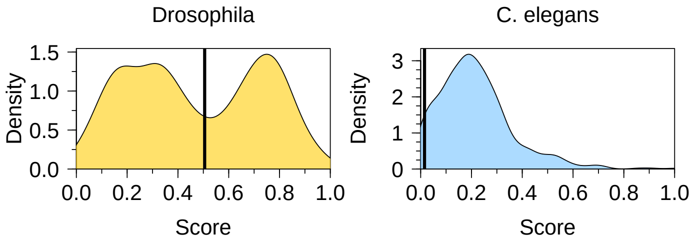

	Ageing implication	Domain conservation	Binding site conservation	Binding affinity	Bioavailability	Lipinski	Promiscuity	Purchasability	Drug approval	**Total**
Drosophila	0.81	0.932	1.0	0.928	(0.9)	-0.2	-0.0	0.0	0.075	0.505
C. elegans	0.81	0.916	1.0	0.928	0.204	-0.2	-0.0	0.0	0.075	0.015

Names

- 4-(10R,11E,14S,18S)-18-(2-amino-2-keto-ethyl)-8,17,20-triketo-14-(1-naphthylmethyl)-7,16,19-triazaspiro5.14icos-11-en-10-ylbenzylphosphonic acid
- 4-(10R,11E,14S,18S)-18-(2-amino-2-oxo-ethyl)-14-(naphthalen-1-ylmethyl)-8,17,20-trioxo-7,16,19-triazaspiro5.14icos-11-en-10-ylphenylmethylphosphonic acid
- 4-(10R,11E,14S,18S)-18-(2-amino-2-oxoethyl)-14-(1-naphthylmethyl)-8,17,20-trioxo-7,16,19-triazaspiro5.14icos-11-en-10-ylphenylmethylphosphonic acid
- 4-[(10s,14s,18s)-18-(2-Amino-2-Oxoethyl)-14-(1-Naphthylmethyl)-8,17,20-Trioxo-7,16,19-Triazaspiro[5.14]Icos-11-En-10-Yl]Benzylphosphonic Acid

Roles

ChEBI entry None has no roles

Status

Approved drug (according to ChEMBL)	No
Classification (according to DrugBank)	experimental
Number of Rule of 5 violations	4
Binding affinity to original target in log units (RF-Score prediction)	7.56
Burns *C. elegans* bioavailability prediction	-7.9

Compound Target Characteristics

Growth factor receptor-bound protein 2

Best gene implication in ageing for this target family came from gene P62994 annotated in UniProt release 2014_02. Annotation GO 7568 (aging) was Inferred from Physical Interaction

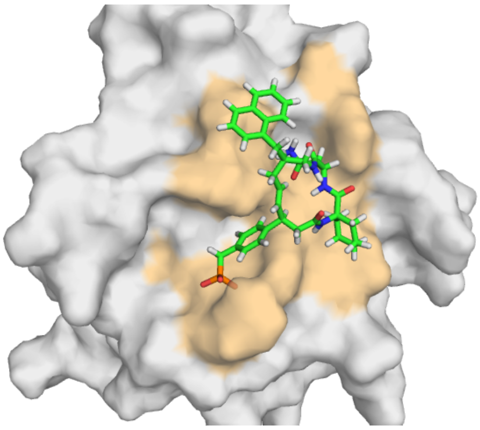

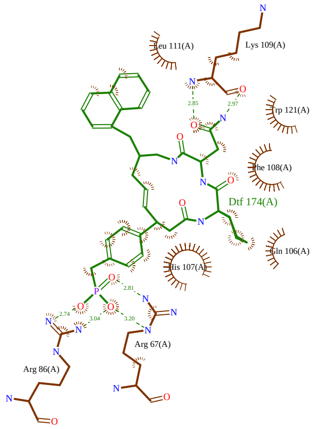

protein	amino acids contacts (binding site)
PDB:1x0n:chainA:P62993	R R Q H F K L W
sp:P62993:GRB2_HUMAN	R R Q H F K L W
tr:B0LPF3:B0LPF3_HUMAN	R R Q H F K L W
sp:P62994:GRB2_RAT	R R Q H F K L W
tr:B1AT92:B1AT92_MOUSE	R R Q H F K L W
sp:Q60631:GRB2_MOUSE	R R Q H F K L W
tr:Q3U5I5:Q3U5I5_MOUSE	R R Q H F K L W
sp:Q08012:DRK_DROME	R R Q H F K L W
sp:P29355:SEM5_CAEEL	R R Q H F K L W

	whole protein		domain-based		contact-based	
protein	ident	simil	ident	simil	ident	simil
PDB:1x0n:chainA:P62993	1.0	1.0	1.0	1.0	1.0	1.0
sp:P62993:GRB2_HUMAN	1.0	1.0	1.0	1.0	1.0	1.0
tr:B0LPF3:B0LPF3_HUMAN	1.0	1.0	1.0	1.0	1.0	1.0
sp:P62994:GRB2_RAT	1.0	1.0	1.0	1.0	1.0	1.0
tr:B1AT92:B1AT92_MOUSE	0.93	0.94	0.97	0.98	1.0	1.0
sp:Q60631:GRB2_MOUSE	1.0	1.0	0.99	1.0	1.0	1.0
tr:Q3U5I5:Q3U5I5_MOUSE	1.0	1.0	0.99	1.0	1.0	1.0
sp:Q08012:DRK_DROME	0.64	0.86	0.64	0.86	1.0	1.0
sp:P29355:SEM5_CAEEL	0.55	0.81	0.56	0.84	1.0	1.0

drk (FBgn0004638) associated phenotypes

dominant, endocytosis defective, fully, learning defective, lethal - all die before end of pupal stage, memory defective, neuroanatomy defective, rescuable maternal effect, some die during pupal stage

(Information from FlyBase)

drk (UniProt:Q08012) annotation
Function: Required for proper signaling by sevenless. May act to stimulate the ability of Sos to catalyze Ras1 activation by linking sevenless and Sos in a signaling complex. (PubMed:8462097, PubMed:8462098).
Subunit: Interacts with autophosphorylated sev via SH2 domain and Sos, Dos and Dab via SH3 domains. Binds to tyrosine phosphorylated Dab via the SH2 domain. (PubMed:12128212, PubMed:8462097, PubMed:8462098, PubMed:9671493).
Subcellular location: Membrane (PubMed:8462098); Peripheral membrane protein (PubMed:8462098).
Tissue specificity: Found mainly in the developing eye and in the antennal disk. Also observed in other imaginal disks tested and in the embryo.

(Information from UniProt)

sem-5 (WBGene00004774) associated phenotypes
cell migration variant, egg laying variant, hermaphrodite sex muscle development variant, maternal effect lethal emb, rod like larval lethal, sex muscle morphology variant, vulval cell induction variant, vulvaless

(Information from WormBase)

sem-5 (UniProt:P29355) annotation
Function: Acts both in vulval induction and sex myoblast migration. Presumably interacts with the kinase receptor let-23 and with a target that modifies the Ras-like protein let-60.

(Information from UniProt)
